# Accelerometry and dual-scale neighborhood indicators for screening of MoCA-defined cognitive impairment: an interpretable machine-learning study

**DOI:** 10.3389/fpubh.2026.1777724

**Published:** 2026-04-02

**Authors:** Lei Zhang

**Affiliations:** Department of Physical Education, Nanjing University of Chinese Medicine, Nanjing, China

**Keywords:** accelerometry, built environment, calibration, cognitive impairment, decision-curve analysis, neighborhood accessibility, older adults

## Abstract

**Background:**

Community-level identification of older adults who may benefit from confirmatory cognitive assessment is constrained by limited resources and the modest accuracy of single-domain screeners.

**Objective:**

This study aimed to develop and internally validate an interpretable prediction model for MoCA-defined cognitive impairment that integrates accelerometry with dual-scale (500 m and 800 m) built-environment indicators, and to evaluate calibration, clinical utility, and subgroup performance. The aim was to support community screening prioritization rather than etiologic inference or clinical diagnosis.

**Methods:**

We analyzed a cross-sectional sample of community-dwelling older adults from Nanjing, China (*n* = 421; March–December 2024). Predictors included accelerometer-derived activity/sedentary metrics, anthropometrics, demographics, and GIS-derived neighborhood accessibility and land-use measures computed within 500 m and 800 m network buffers. Cognitive impairment was defined as MoCA <26. Candidate algorithms (regularized logistic regression, k-nearest neighbors, support vector machine, random forest, and gradient boosting) were trained using stratified 3-fold cross-validation, with an additional stratified 70/30 hold-out test set for internal validation. We reported ROC AUC, precision–recall AUC, Brier score, calibration slope/intercept, decision curve analysis, TreeSHAP-based explainability, and exploratory equalized-odds diagnostics across key subgroups.

**Results:**

Tree-based models achieved the best overall performance. In the held-out test set, random forest showed high discrimination with acceptable calibration (AUC 0.95, 95% CI 0.91–1.00; Brier 0.088), while gradient boosting and support vector machine achieved AUCs approximately 0.90 with lower Brier scores for boosting (0.071). Decision curve analysis indicated a positive net benefit relative to the treat-all and treat-none strategies across clinically plausible risk thresholds. Explainability analyses consistently highlighted MVPA, sedentary time, age, central adiposity, and neighborhood transit accessibility as influential predictors. Subgroup analyses indicated broadly comparable discrimination, with small-to-moderate equalized-odds gaps.

**Conclusion:**

Combining accelerometry with neighborhood indicators may support calibrated, decision-oriented triage for MoCA-defined cognitive impairment in community settings. This model is intended for screening support rather than causal interpretation or diagnostic replacement, and requires external validation before implementation.

## 1 Introduction

Late-life cognitive impairment is common in rapidly ageing cities and often precedes dementia, yet it is frequently under-recognized in routine community health practice (1, 2). In routine community practice, comprehensive neuropsychological assessment is not always feasible at scale, which creates a need for tools that can help identify older adults who may benefit from confirmatory evaluation (3). Accordingly, scalable data-driven risk stratification may support more efficient allocation of limited screening resources. While many studies focus on individual-level predictors, few integrate data from diverse domains such as physical activity, body composition, and the built environment, which may offer a more comprehensive understanding of cognitive risk factors. This study innovates by combining objective accelerometer-derived physical activity data, body composition, and neighborhood built-environment indicators, offering a novel, multi-dimensional approach to predicting cognitive impairment in community-dwelling older adults.

Two modifiable domains are particularly relevant to community screening. First, habitual physical activity and sedentary behavior are linked to cognitive trajectories, but self-reported measures are prone to recall and social-desirability biases (4–7). Hip-worn accelerometers provide objective, high-resolution activity metrics that better capture daily movement patterns (6). Second, the neighborhood’s built environment shapes opportunities for mobility, access to services, and social participation. Geographic information systems (GISs) allow quantification of neighborhood accessibility and land-use patterns that may contribute additional predictive signals beyond individual characteristics (8, 9). Incorporating these environmental factors into predictive models is underexplored in the existing literature, and our study uniquely integrates them to enhance cognitive screening accuracy.

Machine-learning models can integrate multi-domain predictors, but performance reporting in this area often focuses narrowly on discrimination (10, 11). For screening and decision support, calibration (whether predicted risks match observed probabilities), clinical utility (net benefit across reasonable decision thresholds), and equity considerations (whether errors differ across subgroups) are equally important (12–14). Interpretability is also critical if community stakeholders are to trust and act on model outputs (15). This research moves beyond typical performance metrics by emphasizing model interpretability and subgroup fairness, areas often overlooked in similar studies.

Accordingly, the present study focused on screening-oriented risk stratification in an independent 2024 community sample, with emphasis on calibration, decision-curve utility, and subgroup fairness rather than causal explanation. Unlike previous studies that focus on narrow data types or single-domain predictors, our model integrates multiple behavioral and environmental factors, offering a more holistic approach to predicting cognitive impairment. We prespecified an evaluation framework that includes discrimination, calibration, decision curve analysis, and explainability (TreeSHAP), complemented by exploratory subgroup and fairness diagnostics.

## 2 Methods

### 2.1 Study design and participants

We conducted a cross-sectional community study in Nanjing, China, enrolling older residents living independently in the community. Recruitment and all data collection occurred between March and December 2024 after ethics approval was obtained from the Ethics Committee of Nanjing Normal University (approval no. NNU202407010). A multistage, stratified cluster-sampling approach was used. Five urban districts with the highest proportions of older residents were identified from municipal statistics, and 15 communities were randomly selected from district registries. Within each community, approximately 40 residents aged ≥60 years were invited using computer-generated random numbers.

Eligibility criteria were: (1) age ≥60 years, (2) permanent local residence for ≥6 months, (3) independence in basic activities of daily living, and (4) ability to provide written informed consent and complete assessments. Exclusion criteria included a prior diagnosis of dementia, acute stroke within the past 6 months, severe psychiatric disorder, or any condition precluding valid accelerometer wear or cognitive testing. Sampling quotas by age (60–64, 65–69, 70–74, ≥75 years) and sex were set to enhance representativeness; the realized distribution is reported in Supplementary Table S1. After quality control and excluding participants with missing key data (accelerometry or cognitive outcome), 421 participants were retained for analysis. All participants provided written informed consent prior to any study procedures. Reporting followed STROBE, and model reporting followed TRIPOD+AI.

### 2.2 Cognitive outcome and case definition

The target condition was MoCA-defined cognitive impairment at the time of assessment (screening definition, not a clinical diagnosis). Trained staff administered the Chinese version of the Montreal Cognitive Assessment (MoCA) under standardized procedures (3, 16). Consistent with common screening practice, cognitive impairment was defined as MoCA < 26, in the absence of physician-diagnosed dementia and with independence in basic activities of daily living (2, 3). Robustness was assessed by shifting the cutoff by ±1 point; model performance remained robust.

### 2.3 Device-based physical activity measurement

Physical activity was measured using triaxial accelerometers worn on an elastic belt over the right hip for 7 consecutive days during waking hours, with removal during water-based activities and sleep (17, 18). Raw acceleration data were aggregated into 60-s epochs following commonly used ActiGraph processing practices (18). Non-wear was defined using a validated wear/non-wear classification algorithm (≥90 consecutive minutes of zero counts, allowing brief interruptions) (19). A valid day required ≥10 h of wear time, and inclusion required ≥4 valid days, including ≥1 weekend day (18). Counts were categorized as sedentary <100 counts·min^−1^; light 100–1951 counts·min^−1^; and moderate-to-vigorous physical activity (MVPA) ≥ 1952 counts·min^−1^ (17, 18, 20). Daily minutes of MVPA and sedentary time were averaged across valid days. Quality control included device logs, wear-time diagnostics, and visual inspection of time-series plots. Sensitivity analyses confirmed robustness to an alternative valid-day threshold (≥8 h).

### 2.4 Built-environment measurement

Built-environment exposures were derived from municipal geographic information system (GIS) layers time-matched to 2024. Participant community centroids were used as anchor points. Street-network buffers of 500 m and 800 m were delineated using the pedestrian network, consistent with prior GIS-based neighborhood studies in older Chinese adults and multi-scale buffer practices (21, 22). Within each buffer, we computed: (1) intersection density (3-way or higher intersections per km^2^); (2) land-use mix using an entropy index (Shannon entropy ×10; higher indicates greater mix) (23); (3) facility density (per km^2^) for parks/greenways, community fitness venues, primary care clinics, community centers, and public transit stops extracted from the municipal points-of-interest registry; (4) green space ratio (public green/open space area divided by buffer area); and (5) population density (persons per km^2^) harmonized to buffer boundaries. All GIS operations used a consistent projected coordinate system and network-based rather than Euclidean buffers. Indicators were screened for outliers and standardized (z-scores) before modeling. Highly collinear measures (|r| ≥ 0.80) were pruned, retaining the more interpretable indicator.

### 2.5 Other candidate predictors

Additional prespecified predictors included age, sex, years of education, household income, and body composition indices (body mass index and waist–hip ratio). Continuous variables were inspected for skewness and standardized to z-scores; categorical variables were one-hot encoded.

### 2.6 Sample size considerations and feature reduction

The final analytical sample comprised 421 participants, with the number of cognitive-impairment events reported in Supplementary Table S1. To limit overfitting relative to the events-per-variable (EPV) constraint, we used a two-stage reduction strategy. First, we removed near-zero-variance predictors and pairs with excessive collinearity (absolute Pearson’s correlation ≥0.80 or variance inflation factor >5). Second, we applied L1-penalized logistic regression with stability selection across 1,000 bootstrap resamples, retaining predictors with a selection frequency of ≥60%. To further control for complexity, the number of predictors entering tree-based models was capped at 10–12, ensuring EPV ≥ 10 under the main outcome definition. Learning curves were used to confirm that discrimination approached a plateau at the realized sample size.

### 2.7 Data preprocessing and missing data

All preprocessing followed a predefined pipeline. Continuous predictors were inspected for outliers and standardized (z-scores). Covariate missingness was addressed using multiple imputation by chained equations (m = 5) under a missing-at-random assumption. For prediction modeling, imputation was performed within the training data for each resampling split to avoid information leakage; performance metrics were summarized across imputations (see Supplementary material for details). To reduce information leakage, imputation and standardization were fit on the training partition within each cross-validation fold and then applied to the corresponding validation partition; for the 70/30 split, preprocessing was fit on the training set and applied to the held-out test set. Participants missing the outcome or core exposures (accelerometry or cognitive testing) were excluded during cohort curation (Section 2.1).

### 2.8 Model development and algorithms

The task was modeled as binary classification (cognitive impairment vs. normal cognition). We compared a diverse set of algorithms: logistic regression with L2 regularization (transparent clinical baseline), random forest, gradient boosting, XGBoost, and LightGBM. To address class imbalance, we applied class weights inversely proportional to class frequency (and scale_pos_weight where applicable) and constrained tree depth, number of leaves, and learning rate to favor low-variance solutions.

### 2.9 Hyperparameter tuning and cross-validation

Model development used stratified 3-fold cross-validation to compare algorithms and tune hyperparameters; boosting models used early stopping where applicable. The optimization criterion prioritized calibration (the validation Brier score), with ROC AUC as a secondary criterion. To provide a complementary internal estimate of out-of-sample performance, we additionally created a single stratified 70/30 split: models were refit on the 70% training partition and evaluated once on the 30% held-out test set (referred to as the validation set in tables/figures). Because participants were clustered within 15 communities, these internal validation splits were performed at the individual level; future studies should additionally use community-group resampling (e.g., GroupKFold/leave-one-community-out) to better test generalization across neighborhood contexts and reduce the risk of environmental overfitting.

### 2.10 Performance metrics and calibration

The primary discrimination metric was ROC AUC, with 95% confidence intervals estimated by the DeLong method on the held-out test set and by normal approximation of fold-level estimates in cross-validation. Secondary metrics included precision, recall AUC, sensitivity, and specificity at clinically relevant thresholds, and balanced accuracy. Calibration was evaluated using the Brier score, calibration intercept and slope, and loess-smoothed calibration plots. We also computed net reclassification improvement relative to logistic regression.

### 2.11 Decision curve analysis

Clinical utility was assessed using decision curve analysis with thresholds centered on the observed prevalence (prevalence ±20 percentage points). We plotted net benefits for each model alongside treat-all and treat-none strategies, and displayed the difference in net benefit relative to logistic regression, with 95% bootstrap confidence intervals (1,000 resamples). A table of per-threshold net benefits is provided in the Supplementary material to facilitate the audit.

### 2.12 Subgroup analyses and fairness evaluation

We examined transportability and fairness by reporting subgroup performance for sex, age strata (60–69, 70–79, ≥80 years), and education level. For each subgroup, we summarized ROC AUC, calibration slope, and the equalized odds difference (absolute differences in false-positive and false-negative rates). When deviations exceeded a prespecified tolerance of 0.10, we explored threshold adjustments and sample reweighting as sensitivity analyses.

### 2.13 Model explainability

Model explainability was quantified using TreeSHAP on the validation set. To enhance stability relative to the number of outcome events, graphical displays were restricted to the top 10 features ranked by mean absolute SHAP value, and 95% bootstrap confidence intervals for global importance were reported. Partial dependence and accumulated local effects plots illustrated the direction and approximate magnitude of feature–outcome relationships. Stratified SHAP by sex and education is provided in the Supplementary material.

### 2.14 Software, reproducibility, and reporting standards

Analyses were conducted in Python using scikit-learn, XGBoost, LightGBM, and SHAP, with visualization in matplotlib. The analysis plan and codebook were finalized prior to model fitting; all code is version-controlled. Upon acceptance, we will provide de-identified analysis code and a data dictionary under an institutional data-use agreement. Reporting adhered to STROBE for observational studies and TRIPOD+AI for machine-learning–based diagnostic modeling; risk of bias considerations follow PROBAST/PROBAST-AI principles.

## 3 Results

### 3.1 Participant flow and baseline characteristics

**Figure 1:**
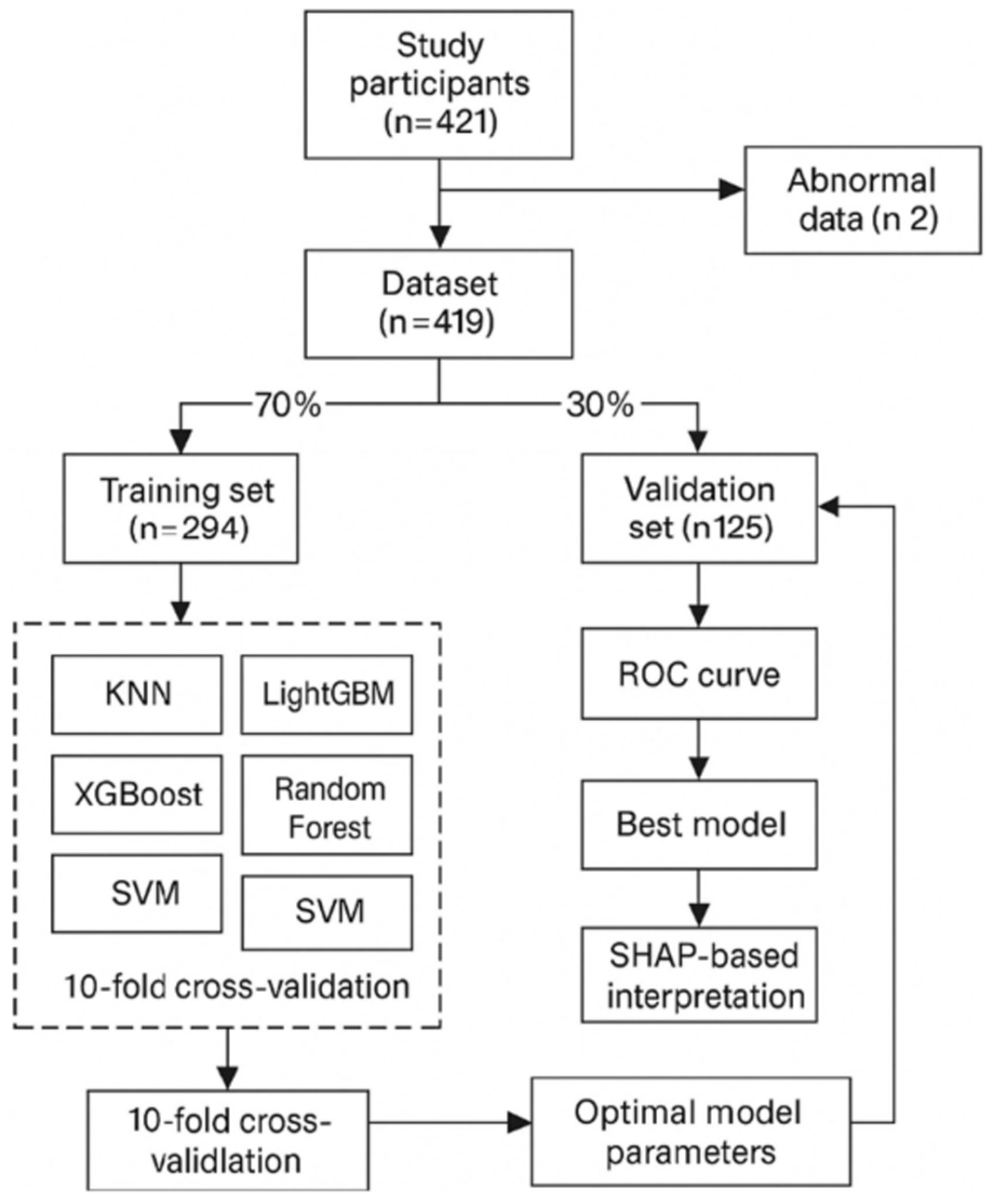
Workflow of participant selection, data preprocessing, model development, and evaluation.

A total of 421 community-dwelling older adults were included in the analytic sample after quality control. The overall prevalence of MoCA-defined cognitive impairment at index assessment and the distribution of key characteristics are summarized in Supplementary Table S1 and Figure 1. Training and validation partitions were created using a stratified 70/30 split. Baseline characteristics for the validation set, together with standardized mean differences (SMDs) vs. the training set, are reported in Table 1.

**Table 1:** Training vs. validation baseline characteristics with standardized mean differences (SMDs).

Variable	Training (*n* = 295)	Validation (*n* = 126)	SMDs
Age (years)	68.46 ± 5.94	68.27 ± 5.96	0.03
Male sex, *n* (%)	116 (39.3%)	58 (46.0%)	0.14
Living alone, *n* (%)	109 (36.9%)	60 (47.6%)	0.22
BMI (kg/m^2^)	25.42 ± 3.40	25.57 ± 3.19	0.04
Waist circumference (cm)	87.55 ± 9.20	88.75 ± 7.87	0.14
Hip circumference (cm)	98.97 ± 6.44	99.69 ± 5.87	0.12
Waist–hip ratio	0.88 ± 0.06	0.89 ± 0.05	0.10
Body fat (%)	29.55 ± 9.57	28.53 ± 8.83	0.11
Muscle rate (%)	66.87 ± 8.99	67.87 ± 8.40	0.12
Grip strength (kg)	28.86 ± 12.21	31.54 ± 15.58	0.19
Chronic disease, *n* (%)	135 (75.4%)	59 (76.6%)	0.03
MoCA score	24.09 ± 3.17	24.15 ± 3.18	0.02
LPA (min/day)	71.39 ± 38.39	66.03 ± 38.19	0.14
MVPA (min/day)	24.81 ± 25.87	26.94 ± 27.93	0.08
Sedentary time (min/day)	588.90 ± 110.05	593.09 ± 116.86	0.04
Facility proximity (m)	122.22 ± 85.64	113.65 ± 86.78	0.10
Number of facilities	4.93 ± 3.67	4.63 ± 3.55	0.08
Number of facility types	3.47 ± 2.26	4.09 ± 2.76	0.24
Facility land area (m^2^)	959.01 ± 1023.01	873.30 ± 971.10	0.09
Population density (per km^2^)	30029.73 ± 19296.16	33051.14 ± 25308.79	0.13
Building density	0.29 ± 0.09	0.30 ± 0.08	0.12
Street connectivity	16.38 ± 4.87	15.78 ± 4.57	0.13
Per capita road length (km)	0.34 ± 0.37	0.28 ± 0.31	0.18
Land use mix	13.42 ± 1.86	13.14 ± 1.91	0.15
Number of transit stations	5.55 ± 2.07	5.55 ± 2.28	0.00
Distance to nearest transit (m)	284.02 ± 116.41	288.24 ± 114.47	0.04
Distance to leisure/entertainment (m)	245.44 ± 161.86	260.77 ± 167.01	0.09
Distance to commercial venue (m)	481.09 ± 264.80	522.07 ± 283.76	0.15

Values are mean ± SD or *n* (%). SMDs are the absolute standardized mean difference; SMDs < 0.10 indicates negligible imbalance. Split: 70/30 stratified by MoCA <26.

### 3.2 Model development and cross-validation performance

**Figure 2:**
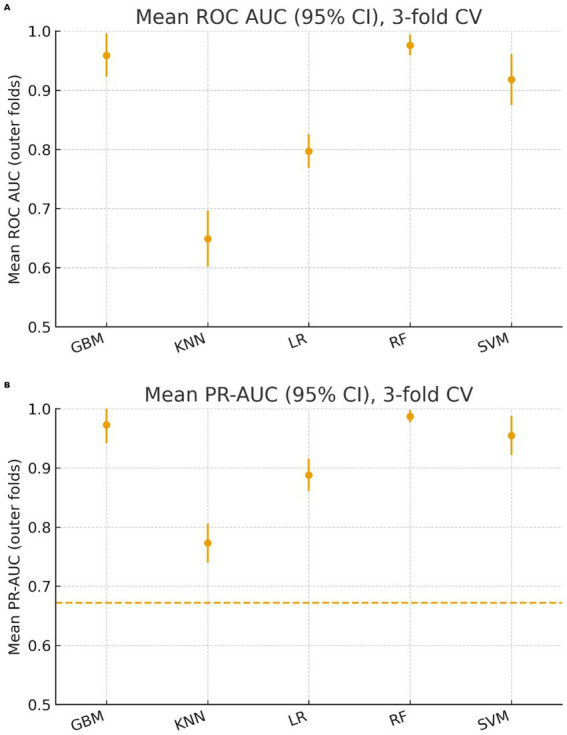
Cross-validated discrimination performance of candidate algorithms. **(A)** Mean ROC AUC with 95% confidence intervals across folds. **(B)** Mean PR-AUC with 95% confidence intervals across folds; the dashed horizontal line denotes the positive-class prevalence (baseline PR-AUC). Points indicate fold-mean estimates; whiskers indicate 95% CIs (normal approximation). CV scheme: stratified 3-fold; outcome: cognitive impairment defined as MoCA < 26. KNN, *k*-nearest neighbors; LR, logistic regression; RF, random forest; SVM, support vector machine (RBF kernel); GBM, gradient boosting machine. Higher values indicate better discrimination; PR-AUC baseline equals prev.

Using stratified 3-fold cross-validation, candidate algorithms exhibited stable discrimination and calibration. Mean ROC AUC and PR-AUC with 95% confidence intervals, together with Brier score and calibration slope/intercept, are summarized in Table 2. Figure 2 visualizes the cross-validated mean AUC and PR-AUC with 95% CIs. Algorithm rankings by joint discrimination–calibration were consistent across folds.

**Table 2:** Model comparison during training: 3-fold cross-validation (mean [95% CI]) (used for algorithm selection and tuning).

Model	ROC AUC	PR-AUC	Brier	Cal slope	Cal int
RF	0.976 (0.959–0.994)	0.987 (0.977–0.998)	0.076	2.36	−0.23
GBM	0.959 (0.923–0.996)	0.973 (0.942–1.000)	0.070	0.91	0.12
SVM	0.918 (0.875–0.961)	0.955 (0.922–0.988)	0.105	1.14	0.28
LR	0.797 (0.769–0.826)	0.888 (0.861–0.915)	0.173	0.57	0.23
KNN	0.649 (0.602–0.697)	0.773 (0.740–0.806)	0.207	0.71	0.20

Stratified 3-fold cross-validation. Confidence intervals are based on normal approximation of the fold-level estimates; for bounded metrics (ROC AUC, PR-AUC), CIs were constrained to the valid range [0, 1]. Outcome: cognitive impairment defined as MoCA < 26.

### 3.3 Internal test-set validation: discrimination and calibration

**Figure 3:**
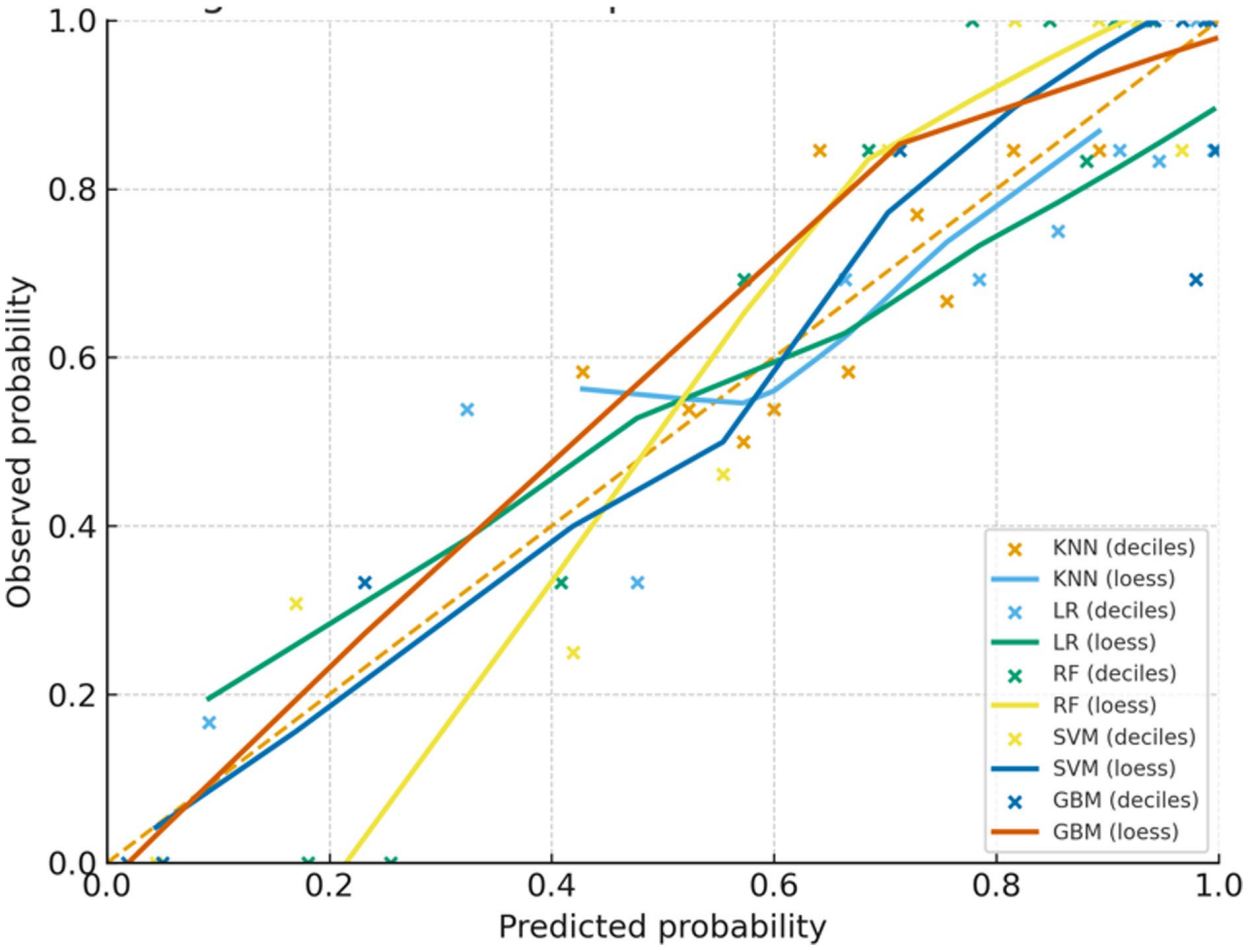
Calibration on the validation set (deciles and LOESS). Decile-based observed event rates (markers) versus predicted probability for KNN, LR, RF, SVM, and GBM. Solid curves are LOESS smooths; the dashed 45° identity line denotes perfect calibration. Brier score, calibration slope, and intercept are reported in Table 3.

Using the fixed 70/30 stratified split, hold-out performance closely mirrored cross-validated estimates. Table 3 reports ROC AUC and PR-AUC with 95% confidence intervals (AUC by DeLong), as well as sensitivity, specificity, and balanced accuracy at thresholds pre-specified on the training set by maximizing Youden’s J. To quantify calibration, Table 3 additionally lists the Brier score, calibration intercept, and calibration slope. Figure 3 shows decile-based reliability plots with LOESS smoothing against the 45° identity line. Algorithm ranking by discrimination was consistent with cross-validation (RF ≈ GBM > SVM > LR > KNN). Calibration was closest to ideal for GBM/SVM, whereas RF produced overly extreme probabilities (calibration slope > 1), indicating overconfidence in high predicted risks. Overall, Brier scores were low for RF/GBM, but probability calibration may be beneficial for deployment.

**Table 3:** Hold-out test performance with discrimination and calibration metrics (validation set, *n* = 126).

Model	AUC (95% CI)	PR-AUC	Brier	Cal slope	Cal int	Sens	Spec	Bal acc
RF	0.953 (0.910–0.996)	0.970	0.088	2.08	−0.13	0.86	0.90	0.88
SVM	0.904 (0.833–0.976)	0.899	0.109	1.04	0.22	0.87	0.88	0.87
GBM	0.898 (0.819–0.977)	0.886	0.071	0.67	−0.03	0.93	0.85	0.89
LR	0.780 (0.690–0.870)	0.843	0.175	0.52	0.13	0.85	0.56	0.70
KNN	0.639 (0.537–0.740)	0.773	0.209	0.70	0.23	0.62	0.56	0.59

AUC 95% CI is based on DeLong; other metrics are point estimates. Thresholds selected on training via cross-validation, maximizing Youden’s J; AUC CI computed by DeLong on the held-out test set.

### 3.4 Clinical utility: decision curve analysis

**Figure 4:**
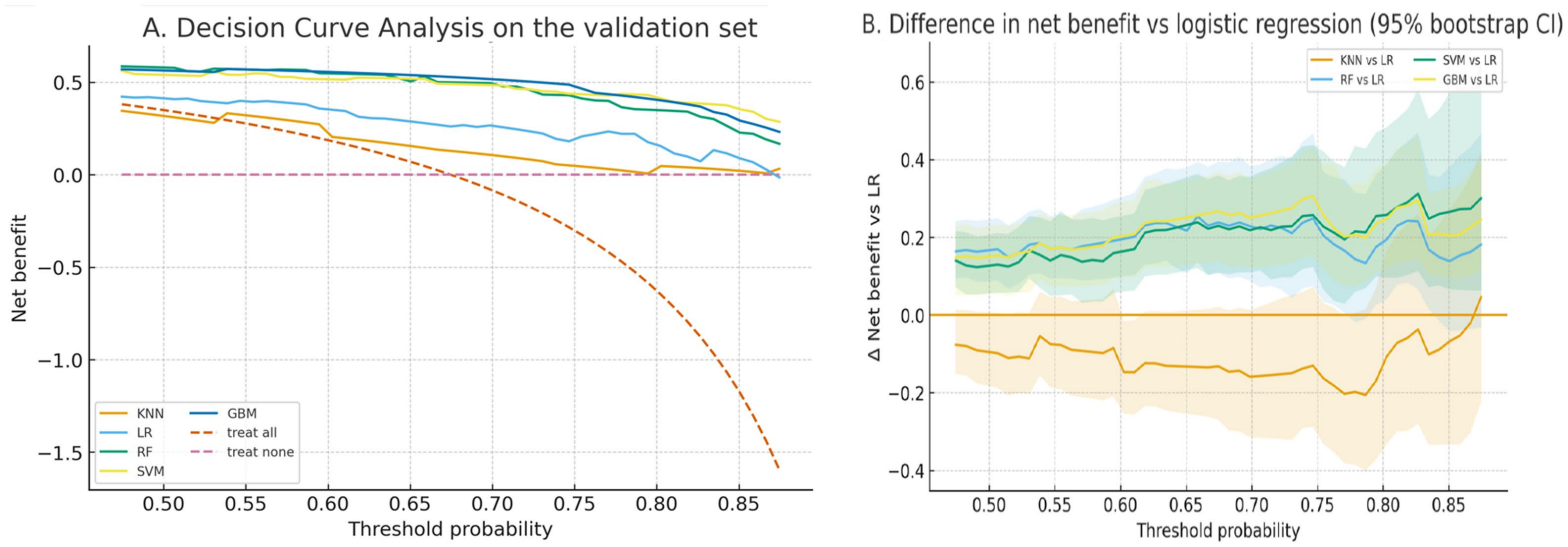
Decision curve analysis on the validation set. **(A)** Net benefit across threshold probabilities centered on the observed prevalence (prevalence ±20 percentage points). Curves are shown for KNN, LR, RF, SVM, and GBM, together with treat-all and treat-none strategies. **(B)** Difference in net benefit (ΔNB) relative to logistic regression with 95% bootstrap confidence intervals. Positive values indicate higher clinical utility than LR at the corresponding threshold.

Decision curve analysis (DCA) quantified net clinical utility over threshold probabilities centered on the observed prevalence (~0.675, i.e., prevalence ±20 percentage points). Figure 4A shows net-benefit curves for all algorithms alongside treat-all and treat-none strategies. To aid comparison, Figure 4B plots the difference in net benefit relative to logistic regression (ΔNB) with 95% bootstrap confidence intervals. Per-threshold net-benefit values are provided in Supplementary Table S2.

### 3.5 Reclassification and parsimony

Relative to the logistic-regression baseline, we quantify category-free net reclassification improvement (cfNRI) on the independent validation set. P(*up/event*) − P(*down/event*) + P(*down/non−event*) − P(*up/non−event*), where “up/down” indicates a higher/lower predicted probability under the non-linear model vs. logistic regression. Table 4 summarizes cfNRI (95% bootstrap CIs) for each non-linear algorithm. SVM and GBM yielded positive reclassification gains vs. logistic regression (SVM: cfNRI = 0.719, 95% CI 0.395–1.048; GBM: 0.899, 0.553–1.230), driven predominantly by improvements among non-events. Random Forest showed a small, CI-crossing improvement; KNN decreased reclassification performance (negative cfNRI).

**Table 4:** Category-free net reclassification improvement (cfNRI) versus logistic regression (validation set).

Model (vs. LR)	cfNRI (95% CI)	Events component	Non-events component
KNN	−0.776 (−1.092–-0.400)	−0.459	−0.317
RF	0.242 (−0.098–0.599)	−0.271	0.512
SVM	0.719 (0.395–1.048)	0.012	0.707
GBM	0.899 (0.553–1.230)	0.435	0.463

cfNRI computed on the validation set; 95% CIs from 400 bootstrap resamples.

To evaluate parsimony, we refit a sparse logistic model using L1-penalization with 5-fold CV on the training set (one-SE preference for sparsity) and then estimated performance on the validation set. The sparse model retained 21 predictors, achieved an ROC AUC of 0.789 (95% CI 0.701–0.877), a PR-AUC of 0.843, a Brier score of 0.167, a calibration slope of 0.88, and an intercept of −0.08, closely matching the full logistic model while substantially reducing complexity (Supplementary Table S3).

### 3.6 Subgroup performance and fairness evaluation

Fairness was evaluated using the Random Forest model (chosen as the primary model based on the highest validation AUC and acceptable calibration) with logistic regression as the baseline. Subgroup analyses by sex, age (60–69, 70–79, ≥80 years), and education are summarized in Table 5, reporting ROC AUC (95% CI, DeLong where estimable) and calibration slope; estimates are suppressed as NA for strata with very small samples.

**Table 5:** Subgroup discrimination and calibration for the primary model (random forest, validation set).

Attribute	Subgroup	*n*	ROC AUC	Calibration slope
Sex	Male	58	0.942	1.818
Sex	Female	68	0.975	2.138
Age	60–69	77	0.990	2.576
Age	70–79	45	0.868	1.288
Age	≥80	4	NA	NA
Education	Primary or below	25	0.727	0.699
Education	Junior high	45	0.960	1.809
Education	High school	43	0.987	2.355
Education	College or above	13	NA	NA

Subgroups with n < 20 are reported as NA due to instability. Validation set: *N* = 126 (events = 85, non-events = 41). 95% CIs by bootstrap (resamples ≥1,000). Results are exploratory due to the limited number of non-event counts.

Equalized-odds disparities were quantified at a global threshold chosen on the training set via Youden’s J. Supplementary Table S4A lists the maximum absolute differences in FPR and FNR across subgroups (optionally with bootstrap 95% CIs). To explore mitigation, we tuned subgroup-specific thresholds on the training set to match overall (TPR, FPR) and evaluated them on the validation set; the resulting changes in disparities and balanced accuracy are reported in Supplementary Table S4B. In our data, sex-related disparities were small; age- and education-related disparities were larger, partly reflecting small or imbalanced strata. Threshold adjustments reduced some gaps but incurred modest accuracy trade-offs, suggesting that reweighting or additional sampling would be preferable for durable mitigation.

### 3.7 Explainability and feature effects

**Figure 5:**
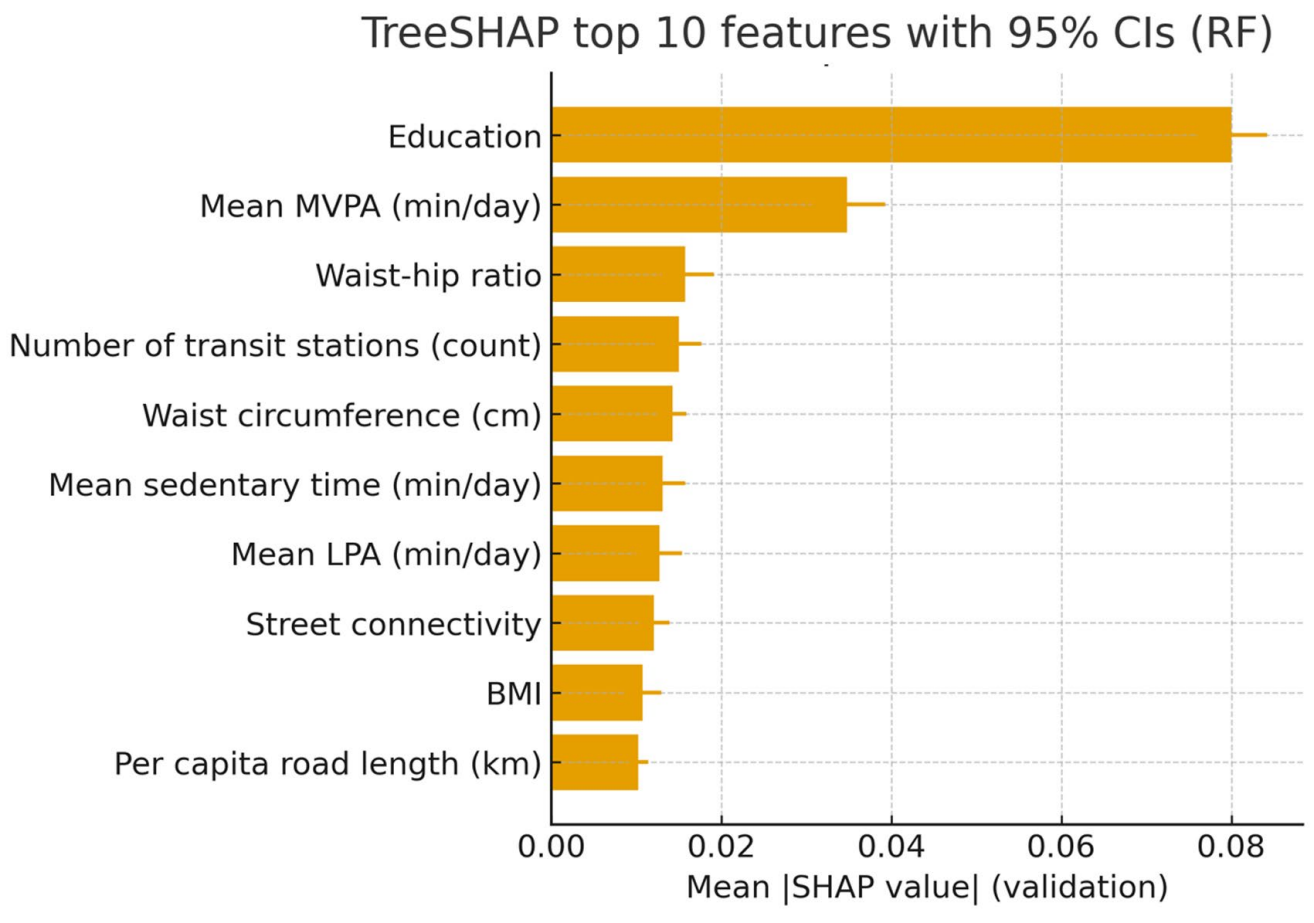
Global feature importance by TreeSHAP on the validation set (random forest). Bars show mean absolute SHAP values; whiskers denote 95% bootstrap CIs (*B* = 500).

**Figure 6:**
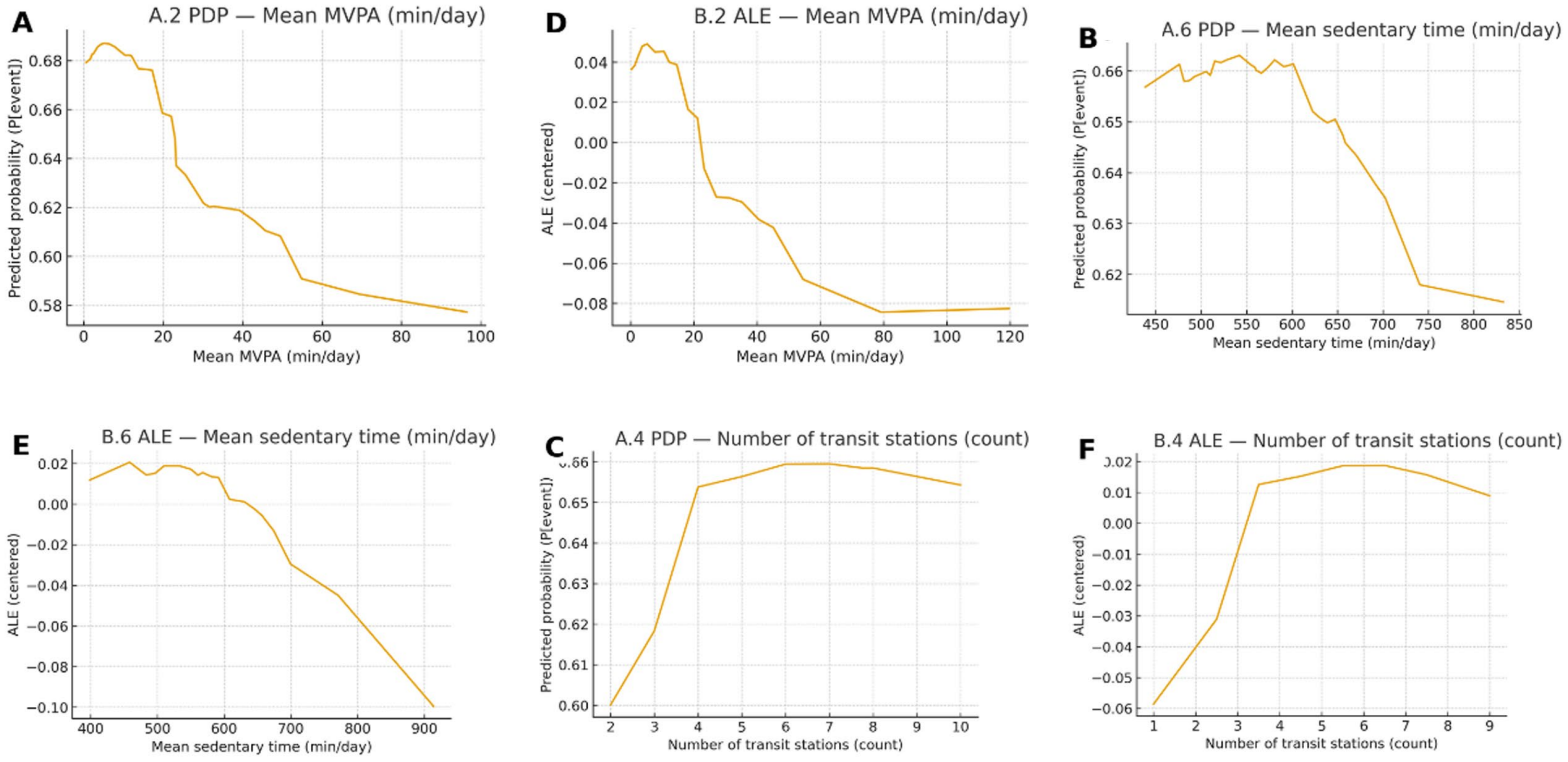
Partial dependence **(A–C)** and accumulated local effects **(D–F)** on the validation set for key predictors (random forest). PDP lines show the average predicted probability across a 5–95% quantile grid; ALE curves are first-order, 20-bin, centered at zero-mean.

Global feature contributions were quantified using TreeSHAP on the validation set with the Random Forest model (primary model). Figure 5 displays the top 10 predictors ranked by mean absolute SHAP value with 95% bootstrap confidence intervals (B = 500). Figure 6 provides partial dependence (PDP) and accumulated local effects (ALEs) for the top predictors to visualize marginal effects on predicted risk. Stratified SHAP summaries by sex and education are presented in Supplementary Figures S1–S2 to compare contribution patterns across subgroups. Estimates in strata with small sample sizes should be interpreted cautiously.

### 3.8 Sensitivity and robustness analyses

Sensitivity analyses supported the robustness of the main findings. Results were similar when (i) varying the MoCA cutoff by ±1 point, (ii) changing the accelerometer valid-day threshold from ≥10 to ≥8 h, (iii) recomputing built-environment indicators using Euclidean 800 m buffers instead of street-network buffers, and (iv) adopting an alternative feature selection procedure (e.g., RFE or Boruta-style selection). Performance summaries for these analyses are provided in Supplementary Table S5 and Supplementary Figures S4–S6. Learning curves and 0.632 + bootstrap optimism estimates are presented in Supplementary Figure S3.

## 4 Discussion

In this cross-sectional community sample, models combining accelerometer-derived activity, neighborhood indicators, and routine participant characteristics provided internally validated support for screening-oriented risk stratification of MoCA-defined cognitive impairment. Across internal resampling and a held-out test set, tree-based approaches generally outperformed simpler baselines, while calibration and decision-analytic results highlighted the importance of evaluating more than AUC when considering potential screening applications (11–13).

From a decision-support perspective, the net-benefit patterns from decision curve analysis suggest that these models could be useful for prioritizing follow-up cognitive assessment within a range of plausible risk thresholds (12, 13). This is relevant for community programs where the cost of comprehensive assessment is non-trivial and where the balance between missed cases and unnecessary referrals must be made explicit (12, 13). Explainability analyses offered coherent, clinically interpretable signals. Higher MVPA and lower sedentary time were consistently associated with lower predicted risk, in line with evidence linking movement behaviors to cognitive health (1, 8). Age and central adiposity measures were also influential, reflecting established associations with cognitive decline and dementia risk (1, 2). Notably, neighborhood transit accessibility and related built-environment measures contributed additional information beyond individual-level predictors, supporting the premise that contextual factors can refine community-level risk stratification (6, 7). Model interpretability, as assessed by SHAP, further supports transparency in community-facing deployment (14).

Equity-aware evaluation is increasingly recommended for prediction tools intended for population deployment (15, 24). In our exploratory analyses, discrimination was broadly similar across major subgroups, although small-to-moderate differences in equalized odds were observed (15). These findings motivate prospective monitoring and, where necessary, pragmatic mitigation strategies (e.g., subgroup-specific thresholds) during future external validation and implementation studies (15).

Several limitations should be considered. First, the design is cross-sectional and cannot establish causal effects of activity or neighborhood exposures. Second, participants were clustered into a limited number of communities; individual-level splits may partially capture shared neighborhood context, yielding optimistic estimates. Future studies should evaluate transportability using community-grouped resampling and truly external cohorts, consistent with recommendations for prediction model validation and reporting (9–11). Third, GIS indicators depend on available spatial data and may be subject to measurement error or temporal mismatch. Finally, internal validation does not substitute for impact evaluation; whether model-guided stratification improves downstream outcomes remains unknown, underscoring the need for prospective evaluation and benefit–harm appraisal (12, 13). Overall, our findings support the feasibility of a calibrated, interpretable, and decision-oriented screening model that integrates objective activity measurement with neighborhood indicators (11–14). The next steps are external validation, prospective replication, and assessment of feasibility and benefit–harm trade-offs in real-world community workflows (9–13, 15).

### 4.1 Implications and future directions

Future studies should (i) validate these models in independent cohorts and across different cities, (ii) adopt clustered resampling designs that respect the community structure, (iii) preregister evaluation plans that include calibration and net benefit, and (iv) test pragmatic deployment strategies (e.g., low-cost data collection and threshold selection) alongside acceptability to community health workers and older adults (9–13, 15). These findings should be interpreted as support for screening workflow optimization rather than evidence for causal intervention targets.

## 5 Conclusion

An internally validated, interpretable model that integrates accelerometer-derived activity with neighborhood built-environment indicators can support calibrated risk stratification for MoCA-defined cognitive impairment in community-dwelling older adults. These results are intended for screening support rather than causal inference and require external validation and prospective evaluation before routine use.

## Data Availability

The original contributions presented in the study are included in the article/Supplementary material, further inquiries can be directed to the corresponding author.
