# Supplementary material for: Accelerometry and dual-scale neighborhood indicators for screening of MoCA-defined cognitive impairment: an interpretable machine-learning study
Source: Front Public Health. 2026 Apr 2;14:1777724. doi: 10.3389/fpubh.2026.1777724 (PMC13082955; doi:10.3389/fpubh.2026.1777724)

Table S1. Participant characteristics by MoCA-defined cognitive status

| Variable | MoCA-defined cognitive impairment (MoCA <26; n=283) | MoCA-normal cognition (MoCA ≥26; n=138) | Overall (n=421) | SMD |
| --- | --- | --- | --- | --- |
| Age | 68.76 ± 5.97 | 67.67 ± 5.83 | 68.40 ± 5.94 | 0.184 |
| Male sex, n (%) | 98 (34.6%) | 51 (37.0%) | 149 (35.4%) | 0.049 |
| Education, n (%) |  |  |  |  |
| Primary or below | 36 (12.7%) | 2 (1.4%) | 38 (9.0%) | 0.439 |
| Junior high | 94 (33.2%) | 16 (11.6%) | 110 (26.1%) | 0.519 |
| High school | 110 (38.9%) | 48 (34.8%) | 158 (37.5%) | 0.085 |
| College or above | 43 (15.2%) | 72 (52.2%) | 115 (27.3%) | 0.782 |
| Income, n (%) |  |  |  |  |
| 0–1000 RMB | 6 (2.1%) | 0 (0.0%) | 6 (1.4%) | 0.207 |
| 1001–2000 RMB | 17 (6.0%) | 3 (2.2%) | 20 (4.8%) | 0.194 |
| 2001–3000 RMB | 79 (27.9%) | 24 (17.4%) | 103 (24.5%) | 0.251 |
| >3000 RMB | 182 (64.3%) | 111 (80.4%) | 293 (69.6%) | 0.361 |
| Not living alone, n (%) | 256 (90.5%) | 129 (93.5%) | 385 (91.4%) | 0.111 |
| BMI (kg/m²) | 25.69 ± 3.44 | 25.00 ± 3.07 | 25.46 ± 3.34 | 0.208 |
| Waist circumference (cm) | 88.65 ± 8.99 | 86.38 ± 8.31 | 87.91 ± 8.83 | 0.259 |
| Hip circumference (cm) | 99.27 ± 6.25 | 99.03 ± 6.34 | 99.19 ± 6.27 | 0.038 |
| Waist-hip ratio | 0.89 ± 0.06 | 0.87 ± 0.06 | 0.88 ± 0.06 | 0.333 |
| Body fat percentage (%) | 29.57 ± 9.72 | 28.59 ± 8.54 | 29.25 ± 9.35 | 0.105 |
| Muscle rate (%) | 66.89 ± 9.14 | 67.74 ± 8.14 | 67.17 ± 8.82 | 0.096 |
| Grip strength (kg) | 29.70 ± 15.06 | 29.58 ± 8.90 | 29.66 ± 13.35 | 0.009 |
| Chronic disease, n (%) | 134 (47.3%) | 62 (44.9%) | 196 (46.6%) | 0.049 |
| MoCA score | 22.38 ± 2.20 | 27.67 ± 1.35 | 24.11 ± 3.17 | 2.695 |
| LPA (min/day) | 67.14 ± 35.77 | 75.22 ± 42.84 | 69.79 ± 38.37 | 0.211 |
| MVPA (min/day) | 21.11 ± 22.96 | 34.35 ± 30.77 | 25.45 ± 26.49 | 0.514 |
| Sedentary time (min/day) | 585.13 ± 102.23 | 600.46 ± 129.58 | 590.16 ± 112.01 | 0.137 |
| Facility proximity (m) | 119.64 ± 88.66 | 119.69 ± 80.48 | 119.66 ± 85.97 | 0.001 |
| Number of facilities | 4.66 ± 3.55 | 5.20 ± 3.78 | 4.84 ± 3.63 | 0.149 |
| Number of facility types | 3.57 ± 2.44 | 3.84 ± 2.42 | 3.66 ± 2.43 | 0.111 |
| Facility land area (m²) | 891.56 ± 953.19 | 1019.08 ± 1108.97 | 933.36 ± 1007.37 | 0.127 |
| Population density (persons/km²) | 32,422.49 ± 22,850.61 | 27,881.52 ± 17,337.78 |  |  |
| Building density | 0.30 ± 0.08 | 0.29 ± 0.10 | 0.30 ± 0.09 | 0.115 |
| Street connectivity | 16.20 ± 4.65 | 16.20 ± 5.06 | 16.20 ± 4.78 | 0.000 |
| Per capita road length (km) | 0.31 ± 0.34 | 0.36 ± 0.37 | 0.33 ± 0.35 | 0.143 |
| Land use mix | 13.39 ± 1.85 | 13.22 ± 1.92 | 13.33 ± 1.87 | 0.091 |
| Number of transit stations | 5.70 ± 2.04 | 5.23 ± 2.29 | 5.55 ± 2.13 | 0.221 |
| Distance to nearest transit (m) | 279.62 ± 111.37 | 296.88 ± 123.75 | 285.28 ± 115.71 | 0.149 |
| Distance to leisure/entertainment (m) | 250.02 ± 161.74 | 250.04 ± 167.27 | 250.03 ± 163.37 | 0.000 |
| Distance to commercial venue (m) | 495.84 ± 274.62 | 488.26 ± 264.10 | 493.36 ± 270.93 | 0.028 |

Notes: Values are mean ± SD or n (%). Overall statistics are derived from group summaries. SMD denotes absolute standardized mean differences.

Table S2. Decision curve analysis net benefit by threshold probability (validation set)

| **p(thr)** | **KNN** | **LR** | **RF** | **SVM** | **GBM** | **treat_all** |
| --- | --- | --- | --- | --- | --- | --- |
| 0.475 | 0.345 | 0.422 | 0.586 | 0.562 | 0.570 | 0.381 |
| 0.483 | 0.337 | 0.417 | 0.584 | 0.544 | 0.568 | 0.371 |
| 0.491 | 0.328 | 0.419 | 0.582 | 0.542 | 0.566 | 0.361 |
| 0.499 | 0.319 | 0.414 | 0.580 | 0.540 | 0.564 | 0.351 |
| 0.507 | 0.310 | 0.408 | 0.578 | 0.538 | 0.562 | 0.340 |
| 0.515 | 0.300 | 0.411 | 0.560 | 0.536 | 0.560 | 0.330 |
| 0.523 | 0.290 | 0.398 | 0.557 | 0.534 | 0.557 | 0.318 |
| 0.531 | 0.280 | 0.392 | 0.573 | 0.558 | 0.555 | 0.307 |
| 0.539 | 0.332 | 0.386 | 0.571 | 0.541 | 0.571 | 0.295 |
| 0.547 | 0.324 | 0.399 | 0.570 | 0.539 | 0.570 | 0.282 |
| 0.555 | 0.316 | 0.394 | 0.568 | 0.548 | 0.568 | 0.269 |
| 0.563 | 0.308 | 0.398 | 0.566 | 0.546 | 0.566 | 0.256 |
| 0.571 | 0.300 | 0.392 | 0.569 | 0.529 | 0.564 | 0.242 |
| 0.579 | 0.291 | 0.386 | 0.568 | 0.528 | 0.562 | 0.228 |
| 0.587 | 0.282 | 0.380 | 0.566 | 0.518 | 0.559 | 0.213 |
| 0.595 | 0.272 | 0.358 | 0.549 | 0.517 | 0.557 | 0.197 |
| 0.603 | 0.204 | 0.351 | 0.547 | 0.515 | 0.555 | 0.181 |
| 0.611 | 0.197 | 0.344 | 0.545 | 0.514 | 0.552 | 0.164 |
| 0.619 | 0.189 | 0.313 | 0.544 | 0.525 | 0.550 | 0.147 |
| 0.627 | 0.181 | 0.305 | 0.542 | 0.524 | 0.547 | 0.129 |
| 0.635 | 0.173 | 0.303 | 0.540 | 0.522 | 0.544 | 0.109 |
| 0.643 | 0.164 | 0.295 | 0.522 | 0.521 | 0.541 | 0.090 |
| 0.651 | 0.155 | 0.287 | 0.504 | 0.519 | 0.538 | 0.069 |
| 0.659 | 0.145 | 0.279 | 0.533 | 0.518 | 0.535 | 0.047 |
| 0.667 | 0.135 | 0.270 | 0.500 | 0.492 | 0.532 | 0.024 |
| 0.675 | 0.129 | 0.261 | 0.499 | 0.490 | 0.528 | 0.000 |
| 0.683 | 0.122 | 0.268 | 0.498 | 0.488 | 0.525 | -0.025 |
| 0.691 | 0.115 | 0.258 | 0.496 | 0.487 | 0.521 | -0.052 |
| 0.699 | 0.107 | 0.266 | 0.495 | 0.484 | 0.517 | -0.080 |
| 0.707 | 0.099 | 0.256 | 0.478 | 0.482 | 0.512 | -0.109 |
| 0.715 | 0.091 | 0.246 | 0.476 | 0.464 | 0.508 | -0.140 |
| 0.723 | 0.082 | 0.234 | 0.459 | 0.462 | 0.503 | -0.173 |
| 0.731 | 0.073 | 0.222 | 0.433 | 0.451 | 0.498 | -0.208 |
| 0.739 | 0.056 | 0.194 | 0.431 | 0.449 | 0.492 | -0.245 |
| 0.747 | 0.050 | 0.181 | 0.429 | 0.438 | 0.487 | -0.284 |
| 0.755 | 0.044 | 0.207 | 0.412 | 0.435 | 0.465 | -0.326 |
| 0.763 | 0.038 | 0.220 | 0.401 | 0.431 | 0.442 | -0.371 |
| 0.771 | 0.031 | 0.233 | 0.399 | 0.428 | 0.435 | -0.418 |
| 0.779 | 0.023 | 0.221 | 0.365 | 0.436 | 0.428 | -0.470 |
| 0.787 | 0.015 | 0.221 | 0.354 | 0.434 | 0.420 | -0.525 |
| 0.795 | 0.006 | 0.176 | 0.351 | 0.431 | 0.411 | -0.584 |
| 0.803 | 0.047 | 0.154 | 0.348 | 0.412 | 0.402 | -0.648 |
| 0.811 | 0.043 | 0.115 | 0.345 | 0.392 | 0.391 | -0.718 |
| 0.819 | 0.039 | 0.098 | 0.341 | 0.389 | 0.380 | -0.794 |
| 0.827 | 0.035 | 0.072 | 0.313 | 0.385 | 0.368 | -0.877 |
| 0.835 | 0.031 | 0.132 | 0.301 | 0.380 | 0.339 | -0.967 |
| 0.843 | 0.026 | 0.115 | 0.264 | 0.375 | 0.324 | -1.067 |
| 0.851 | 0.021 | 0.088 | 0.227 | 0.354 | 0.292 | -1.178 |
| 0.859 | 0.015 | 0.067 | 0.221 | 0.340 | 0.274 | -1.301 |
| 0.867 | 0.008 | 0.028 | 0.191 | 0.302 | 0.254 | -1.439 |
| 0.875 | 0.032 | -0.014 | 0.167 | 0.286 | 0.231 | -1.595 |

**Table S3. Parsimony: sparse logistic regression vs full LR (validation set)**

| **Model** | **Number of predictors** | **ROC AUC (95% CI, DeLong)** | **PR-AUC** | **Brier score** | **Calibration slope** | **Calibration intercept** |
| --- | --- | --- | --- | --- | --- | --- |
| Sparse LR (L1, C=0.187) | 21 | 0.789 (0.701–0.877) | 0.843 | 0.167 | 0.88 | -0.08 |
| Full LR | - | 0.780 (0.690–0.870) | 0.843 | 0.175 | 0.52 | 0.13 |

**Selected predictors (see Excel for full list):** parse logistic regression tuned via 5-fold CV on the training set (L1 penalty; saga solver). Performance evaluated on the validation set; AUC CIs by DeLong

**Table S4A. Equalized-odds differences (max |ΔFPR|, |ΔFNR|) at global threshold**

| **Model** | **Attribute** | **Max \|ΔFPR\|** | **Max \|ΔFNR\|** |
| --- | --- | --- | --- |
| LR | Sex | 0.136 | 0.026 |
| LR | Age | 0.385 | 0.312 |
| LR | Education | 1.0 | 0.788 |
| RF | Sex | 0.095 | 0.02 |
| RF | Age | 0.154 | 0.28 |
| RF | Education | 0.667 | 0.455 |

Equalized-odds differences are reported as the maximum absolute pairwise differences in FPR and FNR across subgroups; values may be unstable in strata with n<10.

**Table S4B. Fairness mitigation via subgroup-specific thresholds (training-based), RF model**

| **Attribute** | **Baseline Max \|ΔFPR\|** | **Baseline Max \|ΔFNR\|** | **Adjusted Max \|ΔFPR\|** | **Adjusted Max \|ΔFNR\|** | **Balanced accuracy (adjusted)** |
| --- | --- | --- | --- | --- | --- |
| Sex | 0.095 | 0.02 | 0.095 | 0.012 | 0.82 |
| Age | 0.154 | 0.28 | 0.154 | 0.34 | 0.84 |
| Education | 0.667 | 0.455 | 0.667 | 0.5 | 0.8 |

For Table S4B, subgroup-specific thresholds are tuned on the training set to minimize squared deviations from the overall (TPR, FPR); performance and disparities are then computed on the validation set.

| **Scenario** | **Model** | **AUC** | **PR-AUC** | **Brier** | **CalSlope** | **CalInt** | **Sensitivity** | **Specificity** | **BalancedAcc** | **Thr** |
| --- | --- | --- | --- | --- | --- | --- | --- | --- | --- | --- |
| Base (MoCA<26) | LR | 0.757 | 0.832 | 0.182 | 0.478 | 0.168 | 0.741 | 0.732 | 0.736 | 0.757 |
| Base (MoCA<26) | RF | 0.945 | 0.961 | 0.089 | 1.994 | -0.182 | 0.718 | 0.951 | 0.834 | 0.745 |
| MoCA<25 | LR | 0.695 | 0.753 | 0.244 | 0.178 | 0.235 | 0.638 | 0.684 | 0.661 | 0.538 |
| MoCA<25 | RF | 0.961 | 0.970 | 0.100 | 2.158 | -0.041 | 0.754 | 1.000 | 0.877 | 0.705 |
| MoCA<27 | LR | 0.730 | 0.906 | 0.197 | 0.114 | 0.922 | 0.811 | 0.548 | 0.679 | 0.689 |
| MoCA<27 | RF | 0.918 | 0.971 | 0.099 | 1.546 | -0.612 | 0.811 | 0.742 | 0.776 | 0.780 |
| Base (MoCA<26) | LR (MI top-10) | 0.754 | 0.815 | 0.179 | 0.699 | 0.063 | 0.694 | 0.707 | 0.701 | 0.726 |
| Base (MoCA<26) | RF (Top-10) | 0.947 | 0.969 | 0.079 | 1.770 | -0.302 | 0.859 | 0.902 | 0.881 | 0.665 |

**Supplementary Figures S1–S2**


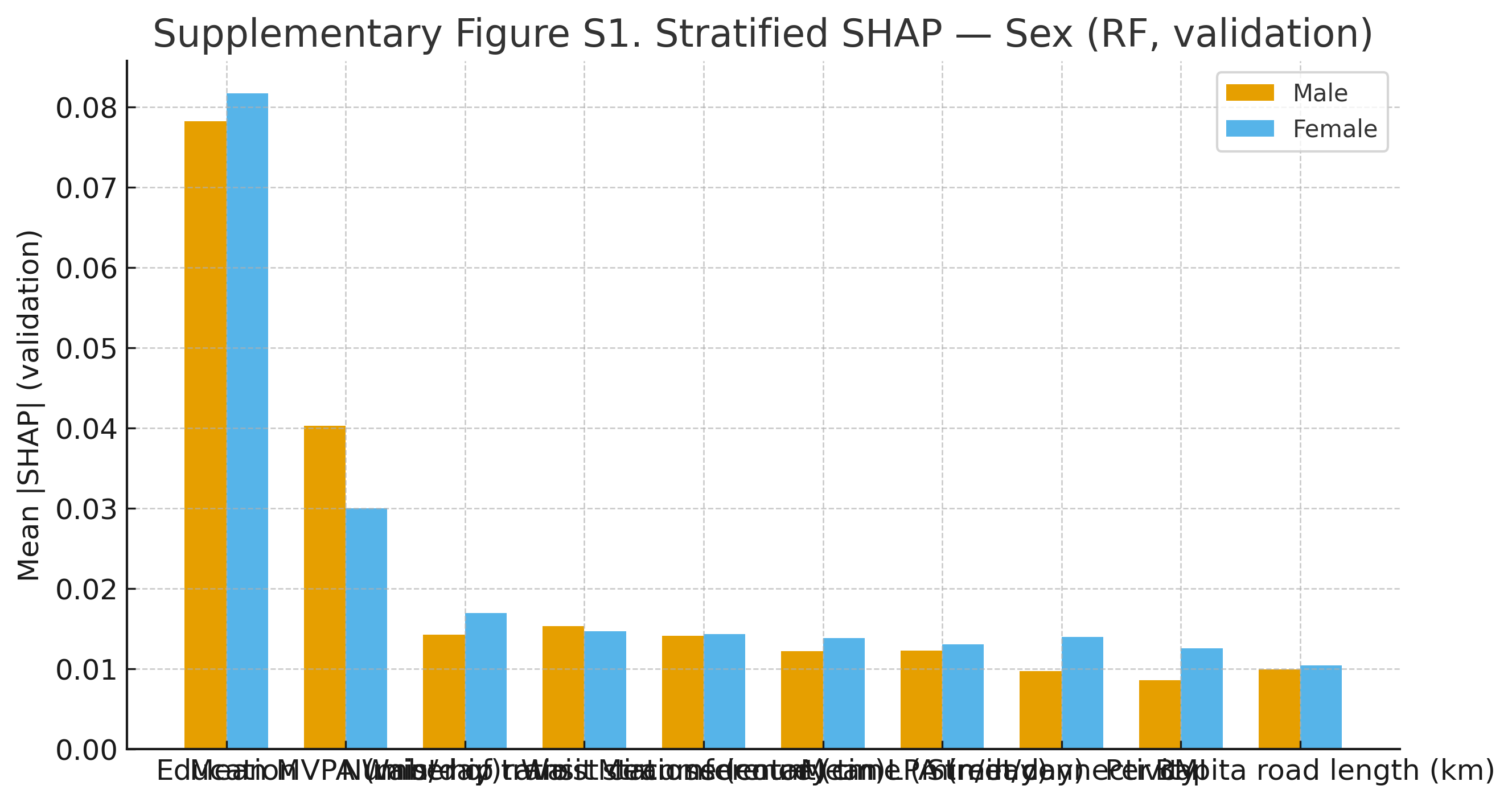


Figure S1. Mean absolute SHAP values on the validation set stratified by sex (Random Forest). Bars compare the top ten predictors across Male and Female groups; results in small strata should be interpreted with caution.


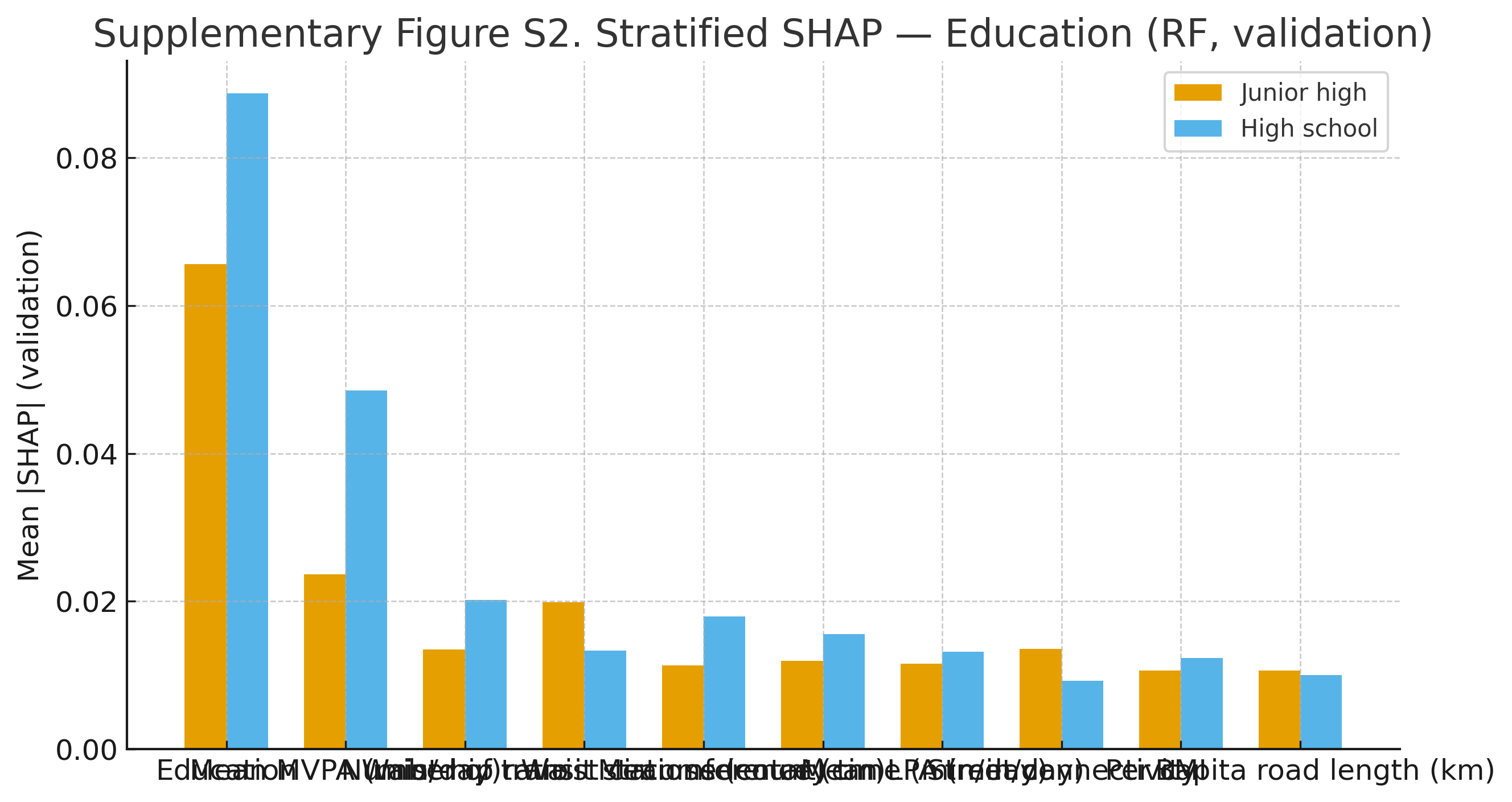


Figure S2. Mean absolute SHAP values on the validation set stratified by education (Random Forest). Bars compare the top ten predictors across the two largest education strata; estimates for small strata are unstable.


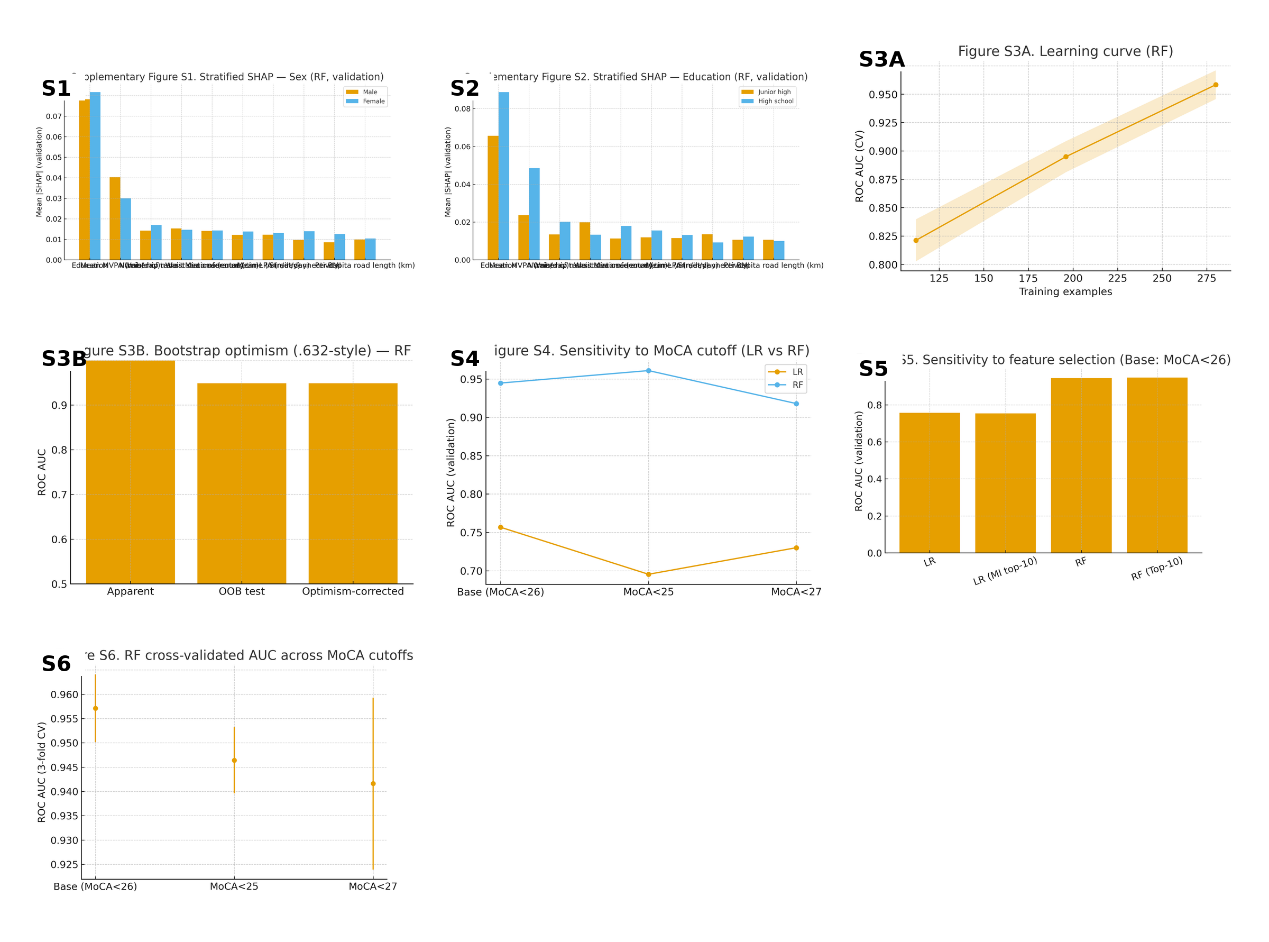

Supplement: Supplementary file 1 [file Supplementary_file_1.docx]
